# Understanding the rise in cardiovascular diseases in Africa: harmonising H3Africa genomic epidemiological teams and tools

**DOI:** 10.5830/CVJA-2014-030

**Published:** 2014-06

**Authors:** Mayowa O Owolabi, George A Mensah, Cristina Rabadan-Diehl, Paul L Kimmel, Rebekah Rasooly, Dwomoa Adu, Michele Ramsay, Salina P Waddy, Bruce Ovbiagele, Sally N Akarolo-Anthony, Charles Rotimi

**Affiliations:** Department of Medicine, University College Hospital, University of Ibadan, Ibadan, Nigeria; Center for Translation Research and Implementation Science, National Heart, Lung, and Blood Institute, National Institutes of Health, Bethesda, MD , USA; Center for Translation Research and Implementation Science, National Heart, Lung, and Blood Institute, National Institutes of Health, Bethesda, MD , USA; Division of Kidney, Urologic, and Hematologic Diseases, National Institute of Diabetes and Digestive and Kidney Diseases, National Institutes of Health, Bethesda, MD , USA; Division of Kidney, Urologic, and Hematologic Diseases, National Institute of Diabetes and Digestive and Kidney Diseases, National Institutes of Health, Bethesda, MD , USA; University of Ghana Medical School, Accra, Ghana; Sydney Brenner Institute for Molecular Bioscience, University of the Witwatersrand; Division of Human Genetics, NHLS and School of Pathology, Faculty of Health Sciences, University of Witwatersrand, Johannesburg, South Africa; National Institute of Neurological Disorders and Stroke, National Institutes of Health, Bethesda, MD , USA; Department of Neurology, Medical University of South Carolina, Charleston, SC, USA; Department of Nutrition, Harvard School of Public Health, Boston, MA , USA; Center for Research on Genomics and Global Health, National Human Genome Research Institute, National Institutes of Health, Bethesda, MD , USA

**Keywords:** cardiovascular diseases, H3Africa, Africa, genomics, epidemiology

## Abstract

**Abstract:**

Cardiovascular diseases, principally ischaemic heart disease and stroke, are the leading causes of global mortality and morbidity. Together with other non-communicable diseases, they account for more than 60% of global deaths and pose major social, economic and developmental challenges worldwide. In Africa, there is now compelling evidence that the major cardiovascular disease (CVD) risk factors are on the rise, and so are the related fatal and non-fatal sequelae, which occur at significantly younger ages than seen in high-income countries. In order to tackle this rising burden of CVD, the H3Africa Cardiovascular Working Group will hold an inaugural workshop on 30 May 2014 in Cape Town, South Africa. The primary workshop objectives are to enhance our understanding of the genetic underpinnings of the common major CVDs in Africa and strengthen collaborations among the H3Africa teams and other researchers using novel genomic and epidemiological tools to contribute to reducing the burden of CVD on the continent.

## Abstract

Non-communicable diseases (NCDs) are currently responsible for over 60% of global deaths. NCDs, which threaten the economic and social development of nations across the globe, are predicted to increase in the coming decades.^1^ Globally, non-communicable cardiovascular diseases (CVDs) are the leading cause of mortality, morbidity and rising healthcare costs.^1^-^5^

Although there are huge data gaps^6^,^7^ and time lags between original data gathering and publication on the current burden of CVD in Africa, recent evidence suggests that the burden of stroke and other CVDs is rising on the continent.^3^,^8^-^12^ Stroke is the second leading cause of death globally, with 70.9% of deaths due to stroke, and 77.7% of the disability-adjusted life years lost occurring in low- and middle-income countries, many of which are in Africa.^8^

Whereas ischaemic heart disease tops the list of CVDs in high-income countries, stroke predominates in African countries.^3^,^10^-^13^ This is possibly because most African countries are in stage two or three of their epidemiological transition.^14^ This epidemiological transition is due to a combination of lifestyle and dietary changes, urbanisation, and demographic transition (increasing life expectancy and population growth),^7^,^14^,^15^ against a background of unique patterns of genomic variation.

Whereas from 1990 to 2010, the age-standardised incidence of stroke significantly decreased by 12% in high-income countries due to the successful deployment of public health tools and interventions,8 it has increased in Africa.^8^,^9^ There is an urgent need to identify knowledge gaps and propose a synergistic research agenda to accurately determine the current burden and fully characterise and quantify the factors underlying this epidemic in Africa.

The Cardiovascular Working Group of H3Africa therefore aims to explore this rising burden of CVDs using novel genomic and epidemiological tools to inform appropriate interventions for the continent. We seek to comprehensively characterise the genomic, sociocultural, economic and behavioural risk factors leading to the development of clinical risk factors (e.g. hypertension and diabetes) and sub-clinical disease (e.g. cardiac and cerebral vascular structural changes), which in turn result in multiple organ damage^7^,^16^ (e.g. stroke, and kidney and heart failure).

To begin this work, the H3Africa Cardiovascular Working Group will hold an inaugural workshop on 30 May 2014 in Cape Town, South Africa, in conjunction with the fourth H3Africa consortium meeting (www.h3africa.org/9-news/125-fourthh3africa-consortium-meeting). The specific working group objectives are (1) to review the current burden of CVDs and their risk factors in Africa, identifying knowledge gaps; (2) to provide the research and surveillance pillar of the integrated CVDs quadrangle (the remaining three pillars are prevention, acute care and rehabilitation) aimed at reducing the rising burden of CVDs; (3) to develop, nurture and strengthen synergistic symbiotic collaboration among H3Africa teams exploring CVDs and their risk factors using novel genomic and epidemiological tools; (4) to enhance our understanding of the genetic underpinnings of the common major CVDs in Africa and across the globe; and (5) to facilitate an appreciation of the pathophysiological interrelationships between cardiometabolic diseases and certain infectious/inflammatory diseases (e.g. rheumatic heart disease, HIV), the heart, brain and kidneys.^7^,^16^-^21^ It is anticipated that refinement and harmonisation of phenotypic characteristics, consideration of environmental factors, and the recruitment of large numbers of well-characterised patients with diverse CVDs across the continent will result in a better understanding of the links between phenotype and genotype in this extremely important group of diseases.

In understanding these complex interrelationships throughout the course of life, we will study important pathways, including endothelial dysfunction, indices of microvascular damage (e.g. albuminuria), oxidative stress, inflammatory processes, as well as endocrine and paracrine influences.^22^-^24^ Furthermore, we will explore how these indices inform the development of riskprediction models and integrated surveillance systems, as well as tailored (responsive) systems and individual-level preventive and therapeutic interventions within and beyond the continent. The H3Africa Cardiovascular Working Group welcomes the active participation of all interested scientists in the inaugural workshop on 30 May 2014 in Cape Town.
